# Changes in Soluble Serum CD81 Concentration during an Oral Glucose Tolerance Test in Patients with Diabetes Mellitus and Individuals with Normal Glucose Tolerance

**DOI:** 10.3390/diagnostics13233500

**Published:** 2023-11-21

**Authors:** Seon Mee Kang, Jun Choul Lee, Bon Jeong Ku

**Affiliations:** 1 Department of Internal Medicine, Kangwon National University College of Medicine, Chuncheon 24289, Republic of Korea; smkang@kangwon.ac.kr; 2 Department of Internal Medicine, Eulji University School of Medicine, Daejeon 35233, Republic of Korea; iamjunchoul@gmail.com; 3 Department of Internal Medicine, Chungnam National University College of Medicine, Daejeon 35015, Republic of Korea

**Keywords:** diabetes, soluble cluster of differentiation 81 (CD81), β-cells

## Abstract

Aim: Cluster of differentiation 81 (CD81) is a cell surface protein involved in cell development, activation, growth, and motility. Recent studies have suggested that CD81 is a marker of dedifferentiated β-cells under conditions of metabolic stress, such as progressive diabetes. However, the clinical significance of changes in soluble serum CD81 (sCD81) in diabetic individuals remains unknown. The aim of this study was to investigate whether serum sCD81 concentrations differ between subjects with diabetes and normal glucose tolerance (NGT), and whether sCD81 changes during a 75 g oral glucose tolerance test (OGTT). Materials and methods: We recruited 101 subjects who had completed an OGTT. According to the test results, the participants were divided into diabetes mellitus (DM) and NGT groups. Participants with prediabetes were excluded from the analysis. During the OGTT, sCD81 levels were measured at 0 and 120 min. We compared changes in sCD81 between the groups. Results: In the DM group, soluble sCD81 levels were significantly higher at baseline and 120 min in the OGTT compared with the normal group (0.59 (0.22–1.05) ng/mL vs. 0.25 (0.81–0.67) ng/mL, 0.55 (0.17–0.96) ng/mL vs. 0.21 (0.92–0.78) ng/mL, *p* = 0.006 and 0.029, respectively). The soluble sCD81 levels in the NGT group remained unchanged (*p* = 0.658), while those in the DM group were significantly decreased during the OGTT (*p* = 0.003). Conclusion: Soluble sCD81 levels were elevated in individuals with type 2 diabetes, such that changes in sCD81 were only observed during the OGTT in the DM group. Soluble sCD81 may have potential as a new diagnostic marker for type 2 diabetes.

## 1. Introduction

The global prevalence of diabetes is increasing rapidly. According to the Korean Diabetes Fact Sheet 2022, one in seven adults over age 30 and one in three people over age 65 in Korea have diabetes [1]. Diabetes mellitus is a multifaceted and complex metabolic condition that ultimately results from abnormalities in insulin secretion, action, or both. When pancreatic beta cells fail to secrete enough insulin to overcome insulin resistance, this results in the inability to maintain glucose homeostasis and the development of type 2 diabetes mellitus (T2DM) [2]. Although new classes of drugs and patient-centered management have led to improved outcomes, additional research is needed to identify effective treatments or cures for progressive β-cell dysfunction. 

Cluster of differentiation 81 (CD81) is one of the 33 members of the tetraspanin family of human membrane proteins. It is encoded by the CD81 gene, which is located on the plus arm of chromosome 11 (11q15.5) [3]. CD81 is also known as target of the antiproliferative antibody 1 (TAPA-1) or tetraspanin-28 (Tspan-28) and is a 26 kDa protein that is expressed in the majority of human cell lines [4]. CD81 provides a scaffold for signaling molecules and orchestrates interactions between membrane-associated proteins to initiate signaling cascades that regulate cell adhesion, migration, and invasion [5,6]. In addition, CD81 is a key component of exosomes, particularly in the context of hepatitis C infection [7]. 

A recent study identified CD81 as a novel surface marker of immature, stressed, and dedifferentiated adult mouse and human islet beta cells (β-cells) [8]. CD81 can be found in either soluble or insoluble form, depending on its association with the cell membrane. Several studies have investigated the exosomal serum fraction of soluble serum CD81 (sCD81) associated with hepatitis C virus (HCV) infection [9]. However, soluble sCD81 levels in diabetic patients and healthy individuals have not been comprehensively examined.

To address this in the present study, we evaluate whether sCD81 levels differ between people with diabetes and normal glucose tolerance, and compared the changes in sCD81 levels during a 75 g oral glucose tolerance test (OGTT).

## 2. Methods

### 2.1. Study Design

This study used a non-randomized, prospective observational design. We recruited 101 subjects (41 men; 53.34 year old) who had indications to complete an OGTT from a group of patients who presented to the Department of Endocrinology, Chungnam National University Hospital (Daejeon, Republic of Korea) between March 2015 and December 2016. The test results were used to divide the participants into diabetes mellitus (DM) and normal glucose tolerance (NGT) groups. Individuals with prediabetes were excluded from the analysis. 

Subjects who met the following inclusion criteria were enrolled in the study: aged between 18 and 65 years, no previous type 2 diabetes diagnosis, and no history of medication with oral hypoglycemic agents or insulin treatment. The exclusion criteria were as follows: type 1 diabetes, severe infection, moderate-to-severe renal impairment (estimated glomerular filtration rate [eGFR] < 45 mL/min/1.73 m^2^), severe hepatic dysfunction, history of stroke, myocardial infarction or other serious vascular complications requiring hospitalization, pregnancy or lactation, childbearing potential, and study ineligibility according to the investigators. 

Our study protocol was approved by the institutional review board of Chungnam National University Hospital (CNUH 2014-12-013), and the study was conducted according to good clinical practice and the principles of the Declaration of Helsinki. Written informed consent was obtained from all patients. 

### 2.2. Data Collection

Clinical parameters including weight, height, waist circumference, and blood pressure were measured using standard techniques. Systolic and diastolic blood pressure (SBP and DBP, respectively) were measured using an electronic blood pressure meter (UA-1020; A&D Co., Tokyo, Japan) with the subject in a sitting position. Blood pressure measurements were taken twice at 5-min intervals, and the mean value was used for analysis. The body mass index (BMI) was calculated by dividing weight (kg) by the square of height (m^2^). Waist circumference was measured at the midline between the lower edge of the rib and the iliac crest using a tape measure. 

To measure biochemical markers, blood samples were collected from all participants at baseline (i.e., after an 8-h fast) and 2 h after ingestion of a 75 g oral glucose load. Samples were collected into ethylenediaminetetra-acetic acid and serum clot activator tubes (Becton, Dickinson and Company, Franklin Lakes, NJ, USA) and immediately centrifuged at 3000 rpm× *g* for 10 min at 4 °C. Soluble sCD81 levels were quantified using a sandwich enzyme immunoassay technique with a commercially available ELISA kit designed for the specific detection of CD81 antibodies (Catalog number: MBS931608; MyBioSource, San Diego, CA, USA). Fasting plasma glucose concentrations and those 2 h after the ingestion of the 75 g oral glucose load were analyzed using the hexokinase method. Glycated hemoglobin (HbA1c) was measured using the Variant II Turbo HPLC analyzer (Bio-Rad, Hercules, CA, USA) in a facility at Chungnam National University Hospital, which is a National Glycohemoglobin Standardization Program level II-certified laboratory. Type 2 diabetes mellitus (T2DM) was diagnosed according to the 2014 American Diabetes Association criteria (13). Triglyceride (TG) levels were measured using the glycerol-3-phosphate oxidase peroxide method. High- and low-density lipoprotein cholesterol (HDL-C and LDL-C, respectively) were measured via homogeneous enzymatic assays. Alanine and aspartate aminotransferase (ALT and AST, respectively) levels and serum creatinine (Cr) levels were measured using a TBA 200FR chemistry analyzer (Toshiba Medical Systems, Tokyo, Japan).

Plasma concentrations of insulin and C-peptide were measured via radioimmunoassay (Roche, Penzberg, Germany). Homeostatic model assessment of insulin resistance (HOMA-IR) and homeostatic model assessment of β-cell function (HOMA-β) were calculated as follows:

HOMA-IR = [fasting serum insulin (µU/mL) × fasting plasma glucose (mg/dL)]/405;

HOMA-β = [fasting serum insulin (µU/mL) × 360] [1]/fasting plasma glucose (mg/dL) − 63].

### 2.3. Study Outcomes and Statistical Analysis

The primary endpoint of this study was the change in sCD81 levels during the OGTT. Secondary endpoints were correlations between sCD81 levels and various metabolic parameters. 

Data are expressed as mean ± standard deviation and percentages for normally distributed data. The median (25–75 quartiles) are used to report on non-normally distributed data. The chi-squared test or Fisher’s exact test were used for analyzing categorical variables. To compare sCD81 levels between the DM and NGT groups, we used the Mann–Whitney U test. To identify factors that influenced the laboratory results before and after ingesting the 75 g oral glucose load, we used a paired *t*-test. We evaluated the data distribution using the Shapiro–Wilk test and histograms. The association analysis for sCD81 was performed separately for each group. Finally, to examine the relationship between sCD81 and clinical data, we calculated Pearson’s correlations. *p*-values < 0.05 were considered statistically significant. All statistical analyses were performed using R software (version 4.1.0; R Project for Statistical Computing, Vienna, Austria).

## 3. Results

### 3.1. Participant Characteristics

Of the 125 study participants, nine withdrew from the study during the OGTT. Of the remaining 116 subjects, 66 were diagnosed with diabetes according to the OGTT data (DM group), and 35 were identified as having NGT (NGT group). Of the 15 patients who did not fit into either group, eight had impaired fasting glucose and seven had impaired glucose tolerance. These patients were excluded from the analysis. 

Table 1 shows the clinical characteristics of the study participants. The mean age of the DM and NGT groups was 50.66 ± 14.39 years and 54.76 ± 11.62 years, respectively, with no significant difference between groups. Women outnumbered men in both groups; there were 22 (62.9%) and 38 (57.6%) women in the NGT and DM groups, respectively, with no statistically significant difference in the gender ratio between groups. We found no difference in mean height or blood pressure between groups, but the DM patients had a higher body weight and BMI than the NGT subjects. Significant differences between the two groups were observed in HbA1c, glucose (fasting, 2-h postprandial), insulin (fasting, 2-h postprandial), fasting C-peptide, HOMA-IR, and HOMA-β levels. The TG level was significantly higher, and the HDL-C level was significantly lower, in the DM group compared with the NGT group.

### 3.2. Primary Endpoint

#### 3.2.1. Changes in Soluble sCD81 Concentrations during the OGTT

**Figure 1:**
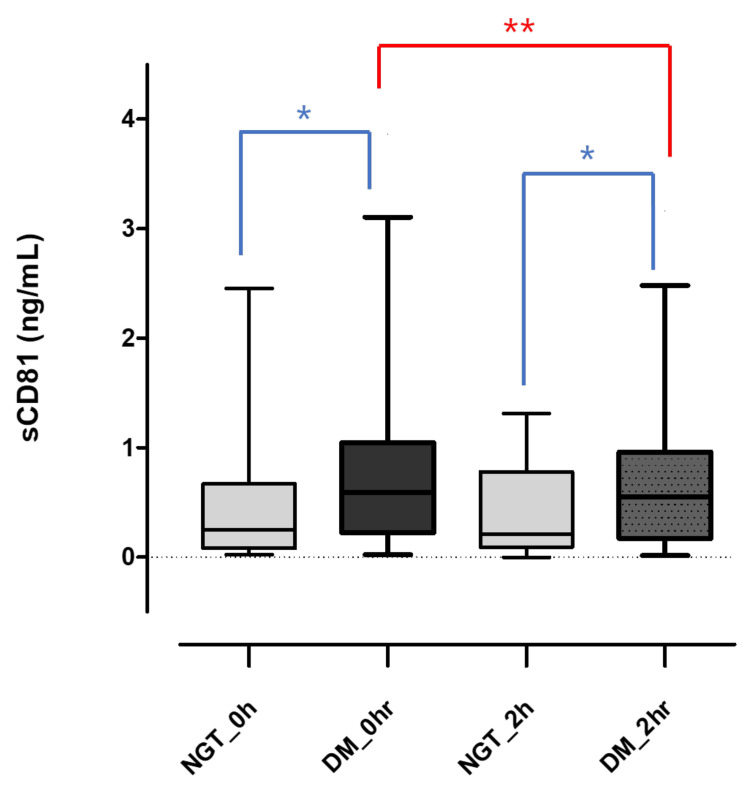
Changes in serum sCD81 concentrations during OGTT in each group. Serum sCD81 levels are shown as the median and (25–75 quartiles). Mann–Whitney U test were used to calculate the *p* values. * *p* < 0.05, ** *p* < 0.01. sCD81, soluble cluster of differentiation 81; 0 h, 0-h concentration during oral glucose tolerance test; 2 h, 2-h concentration during oral glucose tolerance test; NGT, normal glucose tolerance; DM, diabetes mellitus; NGT_0 h, sCD81_0 h in the NGT group; NGT_2 h, sCD81_2 h in the NGT group; DM_0 h, sCD81_0 h in the DM group; DM_2 h, sCD81_2 h in the DM group.

Soluble sCD81 levels were significantly higher in the DM group (0.59 (0.22–1.05) ng/mL) compared to the NGT group (0.25 (0.81–0.67) ng/mL), with a *p* value of 0.006. This difference persisted after 2 h of glucose intake, with values of 0.55 (0.17–0.96) ng/mL vs. 0.21 (0.92–0.78) ng/mL, a significant difference with *p* = 0.029. During the OGTT, sCD81 levels changed significantly in the DM group (*p* = 0.003), whereas there was no significant change in the NGT group (Figure 1).

#### 3.2.2. Soluble sCD81 Concentrations according to HOMA-IR and HOMA-β Status during the OGTT in the DM Group

Statistical differences in sCD81 levels were confirmed in the DM group only during the OGTT. Therefore, we performed additional analyses based on HOMA-IR and HOMA-β status in the DM group. 

**Figure 2:**
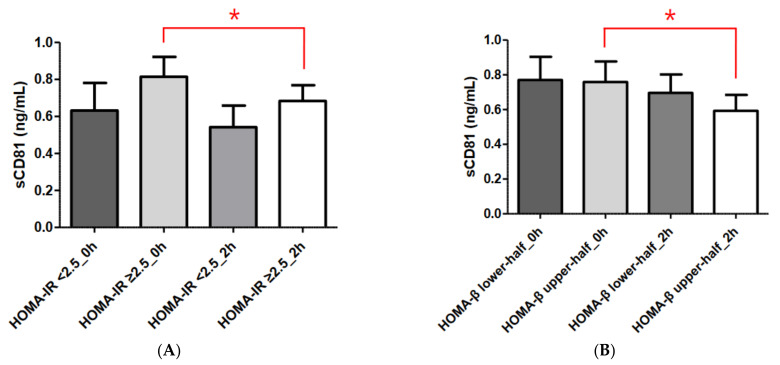
Comparisons of serum sCD81 concentrations according to HOMA-IR and HOMA-β in the DM group. (**A**) Comparisons of serum sCD81 concentrations according to HOMA-IR. (**B**) Comparisons of serum sCD81 concentrations according to HOMA-β. Serum sCD81 levels are shown as the mean ± standard deviation. Student’s *t*-test and the Mann–Whitney U test were used to calculate the *p* values. * *p* < 0.05. sCD81, soluble cluster of differentiation 81; 0 h, 0-h concentration during oral glucose tolerance test; 2 h, 2-h concentration during oral glucose tolerance test. HOMA-IR < 2.5_0 h, sCD81_0 h in the HOMA-IR < 2.5 group; HOMA-IR ≥ 2.5_0 h, sCD81_0 h in the HOMA-IR ≥2.5 group; HOMA-IR < 2.5_2 h, sCD81_2 h in the HOMA-IR < 2.5 group; HOMA-IR ≥ 2.5_2 h, sCD81_2 h in the HOMA-IR ≥ 2.5 group, HOMA-β lower-half_0 h, sCD81_0 h in the HOMA-β lower-half group; HOMA-β upper-half_0 h, sCD81_0 h in the HOMA-β upper-half group; HOMA-β lower-half_2 h, sCD81_2 h in the HOMA-β lower-half group; HOMA-β upper-half_2 h, sCD81_2 h in the HOMA-β upper-half group.

We divided the DM group into HOMA-IR < 2.5 and HOMA-IR ≥ 2.5 subgroups; 18 patients were included in the HOMA-IR < 2.5 group (HOMA-IR = 1.76 ± 0.48) and 48 were included in the HOMA-IR ≥ 2.5 group (HOMA-IR = 4.88 ± 2.18). During the OGTT, the sCD81 levels at 0 and 2 h were higher in the HOMA-IR ≥ 2.5 group than in the HOMA-IR < 2.5 group, but not statistically significantly (sCD81 at 0 h, 0.63 ± 0.63 vs. 0.81 ± 0.74 ng/mL, *p* = 0.360; sCD81 at 2 h, 0.54 ± 0.49 vs. 0.68 ± 0.59 ng/mL, *p* = 0.369). In the HOMA-IR < 2.5 group, we found no significant change in soluble sCD81 levels during the OGTT (from 0.63 ± 0.63 to 0.54 ± 0.49 ng/mL, *p* = 0.33), whereas in the HOMA-IR ≥ 2.5 group, the soluble sCD81 level was significantly reduced during the OGTT (from 0.81 ± 0.74 to 0.68 ± 0.59 ng/mL, *p* = 0.017) (Figure 2A).

The DM group was also divided into HOMA-β upper-half and HOMA-β lower-half groups on the basis of the median HOMA-β value, which was 44.36. Thus, an equal number of patients (*n* = 33) were included in each group (average HOMA-β = 28.24 ± 11.37 and 86.84 ± 52.82, respectively). Soluble sCD81 concentrations were not significantly different between the two groups (sCD81 at 0 h, 0.77 ± 0.76 vs. 0.76 ± 0.68 ng/mL, *p* = 0.947; sCD81 at 2 h, 0.70 ± 0.61 vs. 0.59 ± 0.52 ng/mL, *p* = 0.462). During the OGTT, the sCD81 levels at 2 h were significantly reduced compared to 0 h in the HOMA-β upper-half group (from 0.76 ± 0.68 to 0.59 ± 0.52 ng/mL, *p* = 0.034), whereas we found no significant change in sCD81 levels in the HOMA-β lower-half group (from 0.77 ± 0.76 to 0.70 ± 0.61 ng/mL, *p* = 0.168) (Figure 2B).

### 3.3. Secondary Endpoints: Relationship between Soluble sCD81 Concentration and Various Clinical Parameters

The relationships between various parameters and sCD81 levels at 0 h and 2 h are shown in Table 2 and Table 3, respectively. We observed a significant positive correlation between soluble sCD81 levels at 0 and 2 h when all subjects were analyzed (r = 0.856, *p* < 0.00). There were statistically significant correlations between HbA1c and sCD81 at both 0 and 2 h (r = 0.252 and 0.257, respectively, both *p* < 0.05). In addition, sCD81 at 0 and 2 h tended to be higher in patients with higher 2-h glucose concentrations during the OGTT (r = 0.220, *p* < 0.05 and r = 0.239, *p* < 0.05, respectively). Serum Cr and TG were correlated with sCD81 at 0 h. HDL-C was negatively associated with sCD81 at 0 and 2 h (Table 2).

In the DM group, soluble sCD81 levels at 0 h were associated with those at 2 h (r = 0.857, *p* < 0.001). However, HbA1c and glucose at 2 h were not correlated with sCD81 at 0 or 2 h. Cr and HDL-C were positively and negatively correlated, respectively, with sCD81 at both 0 and 2 h (Table 3).

## 4. Discussion

To the best of our knowledge, this is the first study to compare soluble sCD81 levels during the OGTT between patients with type 2 DM and subjects with NGT. Before and after the OGTT, sCD81 levels were significantly higher in the DM group compared with the NGT group. We found a significant change in sCD81 levels during the OGTT in the DM group but not in the NGT group.

Increased insulin resistance (IR) and decreased insulin secretion comprise the main pathogenic mechanisms underlying T2DM development. When insulin overproduction can no longer compensate for IR, hyperglycemia becomes clinically significant [10]. Impaired β-cell function is a critical requirement for the onset of hyperglycemia and DM, and worsening hyperglycemia is associated with the progressive loss of β-cell function. At the time of diabetes diagnosis, β-cell function is already reduced by more than half and it continues to decline, according to the UK Prospective Diabetes Study (UKPDS) Group [11]. However, the clinical diagnosis of diabetes relies exclusively on elevated blood glucose levels and HbA1c, which are consequences of β-cell function loss rather than β-cell function itself. Therefore, early markers of β-cell function are needed for the diagnosis and pathological management of DM.

IR is strongly linked to cardiovascular risk factors, including T2DM, hypertension, and lipid abnormalities [12]. Although the hyperinsulinemic–euglycemic glucose clamp technique is the gold standard for assessing insulin sensitivity and resistance, HOMA-IR is a widely employed, useful index for assessing IR [13]. A number of studies have used different HOMA-IR cut-off values [14,15,16]. We used a HOMA-IR cut-off value of 2.5 in this study based on the 2008–2010 Korean National Health and Nutrition Examination Survey, which was conducted in a Korean population [17]. In our study, sCD81 levels were higher in the HOMA-IR ≥ 2.5 group (i.e., insulin-resistant group) compared with the HOMA-IR < 2.5 group (i.e., insulin-sensitive group), although not significantly. Furthermore, the sCD81 levels were significantly reduced during the OGTT in the IR group only. These results suggest that sCD81 may be related to IR.

Although both β-cell mass and insulin secretory function are important elements when measuring pancreatic β-cell function, β-cell mass cannot be measured directly in humans. β-cell function can be estimated by measuring insulin or C-peptide secreted with insulin from β-cells. HOMA-β, which is derived from fasting plasma glucose and insulin concentrations, is the most widely used index of insulin secretory function [18,19]. As shown in Figure 2, we found no statistically significant difference in sCD81 level between the HOMA-β lower-half and HOMA-β upper-half groups, although the levels were higher in the former group. In addition, statistically significant changes in sCD81 levels during the OGTT were observed only in the HOMA-β upper-half group. Taken together, these data indicate that sCD81 may be associated with β-cell function.

Healthy pancreatic islets contain both mature and immature β-cell populations. Dedifferentiation, rather than apoptosis, appears to be the primary cause of β-cell failure during metabolic stress and the development of diabetes [20]. CD81 has been identified as a new surface marker of immature, stressed, and dedifferentiated β-cells, while mature β-cells are characterized by the expression of a group of genes related to maturation: Ucn3, Mafa, Glut 2 (in rodents), and Glut 1 (in humans) [8]. In our study, serum levels of sCD81 were significantly higher in the DM group than in the NGT group both before and after the OGTT, and the change in sCD81 levels was significant only in the DM group during the OGTT. In the DM subgroup analysis, we observed a significant change in sCD81 levels during the OGTT only in the groups with HOMA-IR values ≥ 2.5 and HOMA-β values in the upper half of the range. We found significant correlations between sCD81 levels at 0 and 2 h and various metabolic parameters, including HbA1c, 2-h glucose concentrations during the OGTT, TG, and HDL-C, suggesting that the sCD81 level could serve as an indicator of metabolic dysfunction or early stage IR. However, these correlations were not seen in DM patients with persistent IR, poor β-cell function, or hyperglycemia. Taken together, these data suggest that soluble sCD81 may serve as a potential marker of IR and impaired β-cell function, especially in the early stages of these conditions.

There were several limitations to the present study. First, it was conducted at a single institution. Second, the number of subjects was small, which limited the statistical power. A large-scale study is warranted in the future.

## 5. Conclusions

This was the first study to compare soluble sCD81 concentrations between patients with T2DM and subjects with NGT. Soluble sCD81 levels were significantly higher in patients with T2DM compared with individuals with NGT. Changes in sCD81 levels during the OGTT were more pronounced in the DM group, particularly in the high IR and upper half of the HOMA-β range subgroups. These findings suggest that soluble sCD81 levels reflect stress status and pancreatic β-cell function, including insulin secretion, such that they could serve as a potential diagnostic marker for T2DM. 

## Figures and Tables

**Table 1:** Clinical characteristics of the study participants.

	NGT	DM	*p* Value
	(*n* = 35)	(*n* = 66)	
Age (year)	50.66 ± 14.39	54.76 ± 11.62	0.319
Sex, female	22 (62.9)	38 (57.6)	0.763
Height (cm)	163.19 ± 10.02	162.76 ± 9.52	0.833
Body weight (kg)	62.97 ± 10.42	68.00 ± 13.46	0.049
BMI (kg/cm^2^)	23.68 ± 3.77	25.50 ± 3.68	<0.001
SBP (mm Hg)	126.23 ± 13.16	130.58 ± 15.97	0.171
DBP (mm Hg)	76.74 ± 10.83	79.48 ± 10.64	0.224
HbA1c (%)	5.23 ± 0.25	7.48 ± 2.07	<0.001
Glucose_0 h (mg/dL)	92.20 ± 5.30	154.35 ± 58.32	<0.001
Glucose_2 h (mg/dL)	104.74 ± 19.25	284.23 ± 100.46	<0.001
Insulin_0 h (µU/mL)	7.15 ± 3.75	10.81 ± 5.67	<0.001
Insulin_2 h (µU/mL)	36.28 ± 25.51	57.44 ± 49.61	0.040
C-peptide_0 h (ng/mL)	0.56 ± 0.22	0.89 ± 0.38	<0.001
C-peptide_2 h (ng/mL)	2.66 ± 1.23	3.05 ± 1.62	0.270
HOMA-IR	1.64 ± 0.88	4.03 ± 2.33	<0.001
HOMA-β	88.50 ± 45.60	57.54 ± 48.05	0.002
Hb (g/dL)	14.00 ± 1.03	14.23 ± 1.28	0.359
AST (IU/L)	21.53 ± 6.51	23.98 ± 14.66	0.796
ALT (IU/L)	20.53 ± 17.85	26.08 ± 17.97	0.111
BUN (mg/dL)	13.57 ± 3.13	14.30 ± 4.31	0.456
Cr (mg/dL)	0.75 ± 0.14	0.75 ± 0.22	0.469
eGFR	98.86 ± 15.40	106.40 ± 25.05	0.112
TG (mg/dL)	107.64 ± 51.87	165.64 ± 123.30	0.020
TC (mg/dL)	191.56 ± 27.87	195.77 ± 35.62	0.549
HDL-C (mg/dL)	58.58 ± 12.73	51.41 ± 14.08	0.009
LDL-C (mg/dL)	117.48 ± 26.63	119.27 ± 31.06	0.778

NGT, normal glucose tolerance; DM, diabetes mellitus; BMI, body mass index; SBP, systolic blood pressure; DBP, diastolic blood pressure; HbA1c, glycosylated hemoglobin; 0 h, 0-h concentration during oral glucose tolerance test; 2 h, 2-h concentration during oral glucose tolerance test; HOMA-IR, Homeostatic model assessment of insulin resistance; HOMA-β, homeostatic model assessment of β-cell function; Hb, hemoglobin; AST, aspartate transaminase; ALT, alanine transaminase; BUN, blood urea nitrogen; Cr, creatinine; eGFR, estimated glomerular filtration rate; TG, triglyceride; TC, total cholesterol; HDL-C, high-density lipoprotein cholesterol; LDL-C, low-density lipoprotein cholesterol.

**Table 2:** Pearson’s correlations coefficient matrix for variables of all participants.

	sCD81_0 h	sCD81_2 h	BMI	SBP	DBP	HbA1c	Glucose_0 h	Glucose_2 h	ALT	Cr	eGFR	TG	HDL.C	LDL.C
sCD81_0 h	1.000													
sCD81_2 h	0.856 ***	1.000												
BMI	0.106	0.139	1.000											
SBP	0.113	0.134	0.320 ***	1.000										
DBP	0.069	0.052	0.402 ***	0.781 ***	1.000									
HbA1c	0.252 *	0.257 *	0.152	0.172	0.182	1.000								
Glucose_0 h	0.167	0.191	0.227 *	0.133	0.163	0.887 ***	1.000							
Glucose_2 h	0.220 *	0.239 *	0.187	0.162	0.166	0.857 ***	0.841 ***	1.000						
ALT	0.087	0.122	0.532 ***	0.217 *	0.268 ***	0.118	0.147	0.07	1.000					
Cr	0.333 ***	0.347 ***	0.005	0.011	−0.128	−0.012	0.017	−0.033	0.007	1.000				
eGFR	−0.033	−0.008	0.088	0.058	0.152	0.283 ***	0.268 ***	0.324 ***	0.088	−0.604 ***	1.000			
TG	0.214 *	0.143	0.088	0.151	0.17	0.273 ***	0.288 ***	0.309 ***	0.095	0.236 *	0.126	1.000		
HDL-C	−0.244 *	−0.287 ***	−0.246 *	0.035	0.034	−0.258 *	−0.273 ***	−0.315 ***	−0.061	−0.165	−0.071	−0.478 ***	1.000	
LDL-C	0.017	−0.013	−0.015	−0.073	0.031	0.158	0.195	0.164	−0.125	−0.059	0.056	0.085	−0.122	1.000

sCD81, soluble cluster of differentiation 81; 0 h, 0-h concentration during oral glucose tolerance test; 2 h, 2-h concentration during oral glucose tolerance test; BMI, body mass index; SBP, systolic blood pressure; DBP, diastolic blood pressure; HbA1c, glycosylated hemoglobin; ALT, alanine transaminase; Cr, creatinine; eGFR, estimated glomerular filtration rate; TG, triglyceride; HDL-C, high-density lipoprotein cholesterol; LDL-C, low-density lipoprotein cholesterol. * *p* < 0.05; *** *p* < 0.001.

**Table 3:** Pearson’s correlations coefficient matrix for variables of the DM group.

	sCD81_0 h	sCD81_2 h	BMI	SBP	DBP	HbA1c	Glucose_0 h	Glucose_2 h	ALT	Cr	eGFR	TG	HDL.C	LDL.C
sCD81_0 h	1.000													
sCD81_2 h	0.857 ***	1.000												
BMI	0.085	0.126	1.000											
SBP	0.127	0.138	0.281 ***	1.000										
DBP	0.078	0.066	0.421 ***	0.766 ***	1.000									
HbA1c	0.174	0.187	0.032	0.120	0.157	1.000								
Glucose_0 h	0.047	0.081	0.165	0.071	0.141	0.847 ***	1.000							
Glucose_2 h	0.078	0.109	0.005	0.081	0.136	0.817 ***	0.793 ***	1.000						
ALT	0.039	0.057	0.516 ***	0.250 *	0.341 ***	0.071	0.105	−0.052	1.000					
Cr	0.283 *	0.334 ***	0.016	0.047	−0.121	−0.021	0.004	−0.083	−0.071	1.000				
eGFR	−0.130	−0.152	0.085	0.047	0.221	0.285 *	0.258 *	0.370 ***	0.086	−0.669 ***	1.000			
TG	0.208	0.129	0.014	0.067	0.062	0.163	0.186	0.018	0.056	0.279 *	0.136	1.000		
HDL-C	−0.228 *	−0.295 ***	−0.278 *	0.071	0.029	−0.193	−0.209	−0.235	−0.027	−0.199	−0.033	−0.501 ***	1.000	
LDL-C	0.005	−0.006	0.000	−0.112	−0.027	0.184	0.229	0.192	−0.183	−0.117	0.157	0.014	−0.026	1.000

sCD81, soluble cluster of differentiation 81; 0 h, 0-h concentration during oral glucose tolerance test; 2 h, 2-h concentration during oral glucose tolerance test; BMI, body mass index; SBP, systolic blood pressure; DBP, diastolic blood pressure; HbA1c, glycosylated hemoglobin; ALT, alanine transaminase; Cr, creatinine; eGFR, estimated glomerular filtration rate; TG, triglyceride; HDL-C, high-density lipoprotein cholesterol; LDL-C, low-density lipoprotein cholesterol. * *p* < 0.05; *** *p* < 0.001.

## Data Availability

Data is contained within the article.
